# Positive Diagnosis of COVID-19 in an Integrated Teaching and Healthcare Service and Its Associated Factors

**DOI:** 10.3390/healthcare12141395

**Published:** 2024-07-12

**Authors:** Natalia Delgado-Mendoza, Antonella Gómez-Suyón, Ana Miranda-Cárdenas, Roberto A. León-Manco, María Claudia Garcés-Elías

**Affiliations:** Facultad de Estomatología, Universidad Peruana Cayetano Heredia, Lima 15102, Peru

**Keywords:** COVID-19, academic medical centers, health facilities, epidemiological monitoring

## Abstract

Developing and implementing an epidemiological surveillance plan was necessary during the COVID-19 pandemic to ensure safe dental practice. This was due to the high risk faced by this occupational group during the COVID-19 pandemic. This study aimed to determine the factors associated with COVID-19 diagnosis in a Peruvian dental school’s integrated teaching and care service. A cross-sectional study was conducted with a population made up of the records of students, teachers, and administrative personnel in a COVID-19 epidemiological surveillance plan of a dental school during the years 2021 to 2022. The year 2022 was positively associated with a positive diagnosis of COVID-19 (aPR: 1.51; 95% CI: 1.10–2.07; *p* = 0.010) and not having had contact with a patient with COVID-19 was negatively associated with being diagnosed with that disease (aPR: 0.20; 95% CI: 0.14–0.27; *p* < 0.001). In conclusion, 2022 was positively associated with having a positive COVID-19 diagnosis. In addition, not having had contact with a COVID-19 patient was negatively associated with the disease diagnosis and with the development of moderate to severe COVID-19.

## 1. Introduction

COVID-19, whose causative agent is SARS-CoV-2, is a highly contagious disease that caused the last pandemic of the century. It is characterized as asymptomatic or symptomatic and is accompanied by fever, cough, shortness of breath, and gastrointestinal irritation [1]. Consequently, the transmission of the virus posed significant challenges for health professions, including dentistry, being that the practice of dentistry is one of those that represented the most critical risk of contagion due to the exposure to fluids such as saliva and blood, in addition to the necessary proximity to approach patients [2,3]. However, each country showed marked divergences in the attention protocols during the pandemic because epidemiological surveillance and containment plans were scarce up to that point, and the scientific evidence on transmission routes was also limited [4].

During the health crisis, Peru implemented strict measures to prevent and contain the pandemic. Despite these efforts, the country faced the highest number of cases and deaths worldwide. This situation revealed the existing weaknesses in the health system, the informal economy, and the challenging conditions in labor, housing, and transportation [5]. As the strict measures became unsustainable, the government resumed economic activities and ordered the reactivation of the main productive sectors. They named this phase a “new social coexistence,” where workers in specific sectors could gradually return to their jobs, following strict measures to prevent COVID-19 spread [6]. Within this context, the implementation of epidemiological surveillance as a follow-up strategy stands out. This involves observing and gathering important health information from the population, which is subsequently analyzed and interpreted. Its purpose is to detect changes in disease incidence and spread and to identify, measure, and monitor health and disease patterns within the community. This helps understand how diseases and their agents occur and spread, allowing for better responses and interventions [7].

In mid-March 2020, guidelines from various agencies, including the Peruvian government, limited dental care nationwide to urgent or emergency cases only [8,9]. In this context, the Centro Dental Docente (CDD) of the Dental School at Cayetano Heredia Peruvian University (FAEST-UPCH) suspended its services to the public. Despite the challenges and the ongoing risk of contagion among operators, patients, and workers of this integrated teaching and care service, the decision was made to reopen its facilities two months later. This move was part of the broader effort to reactivate economic activities [6].

As part of the efforts to achieve herd immunity, by May 2022, 4.6 billion people worldwide had been vaccinated. This significant milestone aligned with the World Health Organization’s (WHO) objectives, demonstrating the effectiveness of vaccines and people’s commitment to vaccination programs. However, the WHO cautioned that the slowdown in the vaccination pace could become one of the top ten global health threats [10].

Given this context, the resumption of activities at the CDD required a significant commitment from all staff to ensure safe dental practices. Developing and implementing an epidemiological surveillance plan was crucial to understanding and managing the health risks faced by this occupational group. This study aimed to explore the factors associated with COVID-19 diagnosis in the integrated teaching and care service at a Peruvian dental school.

## 2. Materials and Methods

The study design was cross-sectional, retrospective, and analytical, and the population consisted of the records of students, teachers, and administrative staff who participated in the COVID-19 Epidemiological Surveillance Plan (PV-COVID-19) of the CDD of a dental school in a Peruvian university from June 2021 to June 2022. The entire set of records from the mentioned period was considered the population, and sampling was not required. In 2021, there were 754 records, and 654 records for 2022, reaching 1408 records. Regarding the participation criteria, all records of students, faculty, and administrative staff who participated in the CDD PV-COVID-19 for 2021 and 2022 were included. The records that did not contain complete information on the study variables were excluded. It should be noted that the information collected for the survey was taken only from individuals who participated voluntarily. Those absent from work or on medical leave due to illness during the survey period were excluded.

The dependent variable was self-reported COVID-19 diagnosis, defined as the presence or absence of the disease. Other variables were included, such as the year of the survey, which was categorized as 2021 or 2022, and the COVID-19 diagnostic method, classified as clinical suspicion, total serological test (IgG and IgM), antigen test, and molecular test. Other variables also included the type of COVID-19 clinical spectrum grouped into asymptomatic, mild, moderate, and severe; whether vaccinated against COVID-19 (at least two doses); contact with COVID-19 patient; presence of a systemic condition; and nutritional condition according to body mass index (BMI). Also covered was the academic level at which individuals’ work, classified as undergraduate, graduate, and postgraduate; their role, whose possible answers were administrative/assistant staff, student, and faculty; and the place where they work (any of the three CDD locations), provenance, gender, and age.

To begin the study, permission was requested from the Administrative Directorate of the FAEST-UPCH to access the database of the persons surveyed. Subsequently, we proceeded to discard the records that did not have complete information, or that did not meet the research requirements, and then exported the final information to the Microsoft Excel program. The statistical analysis consisted of a descriptive analysis of the variables to obtain relative and absolute frequencies. A bivariate analysis was conducted using the Chi-square test. Finally, a multivariate analysis was performed using Poisson regression to determine crude prevalence ratios (PRs) and adjusted prevalence ratios (aPRs); the latter were selected based on their association in the bivariate analysis. STATA version 17.0 software was used, and the study was conducted with a 95% confidence level and a *p*-value of less than 0.05. One of the authors (RALM) performed the statistical analysis.

Before the project’s execution, approval was obtained on 17 August 2022, from both the Dental School and the Institutional Ethics Committee of the Universidad Peruana Cayetano Heredia (CIE-UPCH), with code SIDISI 209295. It is essential to point out that the database only contains coded records, which prevents participant identification and guarantees anonymity.

## 3. Results

For 2021, 20.56% (n = 155) of the population studied within the epidemiological surveillance plan had a positive COVID-19 diagnosis, while for 2022, it was 30.12% (n = 197). Regarding the method used for diagnosis, in 2021, 35.48% (n = 55) were diagnosed by a total serological test, and by 2022, 41.12% (n = 81) by molecular test. Regarding the type of COVID-19 clinical spectrum, in 2021, 35.48% (n = 55) were asymptomatic, and in 2022, 45.18% (n = 89) belonged to the mild clinical spectrum. In 2021, 52.92% (n = 399) were not vaccinated against COVID-19; by 2022, 94.5% (n = 618) had already been inoculated. In 2021 and 2022, 72.15% (n = 544) and 64.37% (n = 421) had had contact with a patient with COVID-19, respectively. Regarding nutritional condition according to BMI, by 2021, 48.14% (n = 363) had a normal weight; by 2022, this increased to 60.86% (n = 398). According to the academic level at which they work, in 2021, 78.54% (n = 194) worked at the postgraduate level, and by 2022, 72.49% (n = 361) worked at the same level. In 2021, 67.24% (n = 179) of those evaluated were administrative and auxiliary personnel; by 2022, this group decreased to 66.06% (n = 432). According to place of work, in 2021, 77.59% (n = 585) worked exclusively in San Martin de Porres (SMP); in 2022, only 59.02% (n = 386) did so. In 2021, 86.47% (n = 652) were from Lima, 54.38% (n = 410) were male, and 76.39% (n = 576) were adults. On the other hand, in 2022, 94.19% (n = 616) were from Lima, 67.74% (n = 443) were female, and 51.83% (n = 339) were adults (Table 1).

Likewise, in 2021, vaccination against COVID-19, having had contact with a positive patient, being systemically compromised, nutritional condition, work location, provenance, gender, and age were associated with COVID-19 diagnosis. Similarly, the method of diagnosis of COVID-19 was associated with having had contact with a COVID-19-positive patient, systemic and nutritional status, job role, provenance, gender, and age. Finally, having had contact with a patient, having a systemic condition, nutritional condition, work location, provenance, gender, and age were associated with the type of COVID-19 symptoms developed by the individuals (Table 2).

Regarding the year 2022, COVID-19 diagnosis was associated with having had contact with a patient with COVID-19, systemic and nutritional condition, level of study, place of work, provenance, and age. Regarding the method of diagnosis, vaccination, contact with a COVID-19 patient, systemic and nutritional condition, work role, provenance, gender, and age were the variables that showed an association. Meanwhile, vaccination, contact with a patient, systemic condition, academic level, job role, place of work, provenance, and age were associated with the type of COVID-19 clinical spectrum (Table 3).

In multivariate analysis, the year 2022 was positively associated with a positive COVID-19 diagnosis (aPR: 1.51; 95% CI: 1.10–2.07; *p* = 0.010), and not having had contact with a patient with COVID-19 was negatively associated with being diagnosed with the disease (aPR: 0.20; 95% CI: 0.14–0.27; *p* < 0.001). Additionally, not having had contact with a patient with COVID-19 was negatively associated with developing moderate to severe COVID-19 (aPR: 0.48; 95% CI: 0.24–0.96; *p* = 0.036). It should be mentioned that the variables adjusted for this analysis were the academic level at which individuals’ work, role, place, provenance, gender, and age (Table 4).

## 4. Discussion

According to the study’s findings, the year 2022 was associated with a positive COVID-19 diagnosis, compared to 2021. In this regard, before declaring the end of the international health emergency, Peru faced five waves of COVID-19 [11]. The beginning of 2022 was affected by the development of the third wave, characterized by being the least deadly and the shortest of all. In addition, the third wave peaked five times higher than the second. It was characterized by the appearance of the BA.1 omicron variant [12], with significantly higher transmissibility than other variants existing up to that time [13]. Considering all these factors, it is understandable that teachers, students, and workers have a higher probability of testing positive for COVID-19 in that specific year. The increase in the number of positive cases tested at the CDD during the second year of the surveillance plan is a clear indication of the situation.

From 1 March 2020 to 13 June 2023, Peru recorded a cumulative total of 132,376 confirmed cases of COVID-19 per million inhabitants [14]. Additionally, in January 2021, the OPENCOVID-Peru portal reported a national reproduction rate (Rt) of 1.29, indicating that, on average, one infected person could transmit the virus to 1.29 others; however, it was observed that in Lima Metropolitan, where the CDD is located, this rate was 0.93, suggesting a lower likelihood of transmission within that area [15]. Our study revealed that the absence of contact with a COVID-19 patient is negatively associated with disease diagnosis. Analyzing this, it can be observed that although the number of infection cases across Peru was significant, the transmission probability during the period when the CDD had resumed its activities was low, which aligns with the notion that if the number of infected individuals is low, the likelihood of contracting the virus is also low. Another effort that could contribute to controlling the number of infections was the emphasis on wearing masks in crowded places during 2021 and 2022 in Peru. It is important to note that in 2021, this requirement became even stricter, recommending double masks, one of which could be cloth, and the option of using a face shield [16]. However, by October 2022, the use of these measures became optional [17].

About this, a study developed a model to estimate the risk of COVID-19 infection in the most frequent indoor settings such as classrooms, offices, restaurants, and libraries [18]. Viral load, expressed in quanta, is one of the primary factors linked to the likelihood of infection. In addition, specific characteristics are included such as room dimensions; environmental parameters such as temperature, relative humidity, atmospheric pressure, UV index, and CO_2_ concentration; the number of people and the duration of activity; the number of individuals wearing masks and their type; as well as the immune population, whether vaccinated or infected. Among the findings, it is reported that the probability of infection in classrooms, restaurants, and libraries is less than 2% for an exposure time of 2 h; however, this may increase if the exposure time is exceeded, specifically if it is exceeded by 90 min. When it comes to dental care and educational settings, one must consider the exposure of operators to patient aerosols and the concentration of teachers, students, and administrative staff during daily activities. These factors can influence viral load and the likelihood of infection.

Some dental schools worldwide have developed protocols to adapt their practices to a health emergency. Concerning health care education services, a university in Kuwait defined that the return to these environments would be arranged in five stages: the preparatory phase would comprise two months, where they would verify the stock of personal protective equipment, train staff on infection control and disinfection, equipment maintenance, and evaluation of ventilation and water systems. This phase would be followed by Phase 1, where only dental emergencies and emergencies would be attended; in Phase 2, all procedures not involving the emission of aerosols would be performed; Phase 3 would return to pre-COVID-19 operations, allowing students to perform all dental procedures, although ultrasonic scaling and the preparation of prosthetic crowns would require a negative polymerase chain reaction test. Finally, Phase 4 would be scheduled to begin at the end of the pandemic.

A contact tracing team was also implemented as part of the protocol, and as of 20 May 2021, they identified 26 confirmed cases of COVID-19, including academics, dental assistants, and students. It was noted that the interactions of the positive cases in the dental school did not meet the definition of “close contact”, which involved spending more than 15 min within 2 m of an infected person without the proper use of protective equipment. Also, 32 participants agreed to have close contact off-campus [19]. Unlike the Kuwaiti case, FAEST-UPCH started clinical activities in 2021, hand in hand with its epidemiological surveillance plan, where case tracking among administrative staff, students, and faculty was a substantial part.

Another published example of the experiences of university dental clinics and their performance during the pandemic was reported by Lingawi et al [20]. This center located in Saudi Arabia saw more than 1500 patients between September 2020 and March 2021; however, less than 4.5% of its workers contracted COVID-19 during that period. On tracing, it was identified that infections were acquired at the community level and that no virus was transmitted to students, hospital staff, or patients.

On the other hand, the present research identified that not having had contact with a patient with COVID-19 was negatively associated with developing moderate to severe disease. Accordingly, an analysis of the evolutionary characteristics of SARS-CoV-2 subtypes suggests that mutations that emerged after May 2021 were beneficial for the virus and probably improved its transmission capacity, immune evasion, and infection, among others [13]. The main variants of this virus are Alpha, Beta, Gamma, Delta, and Omicron, and their transmissibility has been progressively increasing over time, with Gamma and Delta standing out as the most clinically aggressive. In early January 2021, the World Health Organization (WHO) designated Gamma as a variant of concern (VOC) due to its increased transmissibility. Although an increase in deaths was initially reported in Brazil, the country from which the first detected cases originated, an increase in hospitalizations was reported in Europe. It is explained that the situation in Brazil may have been triggered by the collapse of the health system and socio-economic constraints in the country. In the same year, the Delta variant emerged, also classified as a VOC, with a high infectious capacity and a higher rate of adverse outcomes in those infected, such as hospitalizations, intensive care unit (ICU) requirements, and deaths [21].

Despite this scenario, in October 2020, the Peruvian Ministry of Health established the National Vaccination Plan against COVID-19, where a prioritization was defined according to vaccine availability, organized in three phases: the first to protect health professionals and ensure the continuity of essential services, the second to reduce morbidity and mortality in the population at the most significant risk, and the third to generate herd immunity [22]. On 5 April 2021, vaccination began for Peruvian dentists in the country’s capital [23] and subsequently for the general population according to age groups and in decreasing order (from oldest to youngest) [24]. According to the Johns Hopkins University Coronavirus Resource Center, 88,062,495 vaccine doses were administered in Peru, with 92.04% of the general population receiving at least one dose [25]. This indicates a broad acceptance of COVID-19 immunization during the early years of the pandemic, resulting in a reduced likelihood of both contracting the infection and consequently developing moderate to severe disease while favoring the resumption of pre-pandemic activities such as dental care in a CDD.

One limitation of this study is the small sample size, with 755 records in 2021 and 648 in 2022. It is recommended that future research be carried out with a larger sample. Additionally, although appropriate for health sciences research, the longitudinal design of the study implied the loss of participants between the two years due to its long duration. It should be mentioned that since this is a cross-sectional study, causality in the results cannot be established.

This study addresses the identification of factors associated with a positive COVID-19 diagnosis in an integrated teaching and care service, given the high risk of contagion in this type of setting due to the proximity between the operator and the patient during procedures. Therefore, it was necessary to design, implement, and evaluate an epidemiological surveillance plan that included the tracking and follow-up of positive cases during the clinical practice of dental training. In the future, the main benefit of this research will lie in the ability to act and control the risk of contagion in future epidemics or pandemics that challenge the performance and teaching of professions such as dentistry.

## 5. Conclusions

Within the framework of an epidemiological surveillance plan carried out in a Peruvian dental school’s integrated teaching and care service, factors associated with a positive diagnosis of COVID-19 were identified. The study revealed a positive association between the year 2022 and the diagnosis of COVID-19. It was also found that individuals who did not come into contact with COVID-19 patients showed a negative association with a positive disease diagnosis or development of moderate to severe cases.

## Figures and Tables

**Table 1:** Characteristics of the COVID-19 epidemiological surveillance plan population in an integrated teaching and care service.

Variables		Year				
		2021		2022		*p* *
		n	%	n	%	
COVID-19 diagnosis						
	Yes	155	20.56	197	30.12	<0.001
	No	599	79.44	457	69.88	
Method of COVID-19 diagnosis						
	Clinical suspicion	23	14.84	39	19.8	<0.001
	Serological test	55	35.48	32	16.24	
	Antigen test	23	14.84	45	22.84	
	Molecular test	54	34.84	81	41.12	
Type of COVID-19 clinical spectrum						
	Asymptomatic	55	35.48	52	26.4	0.182
	Mild	54	34.84	89	45.18	
	Moderate	45	29.03	54	27.41	
	Severe	1	0.65	2	1.02	
COVID-19 vaccine (at least two doses)						
	Yes	355	47.08	618	94.5	<0.001
	No	399	52.92	36	5.5	
Contact with COVID-19 patient						
	Yes	210	27.85	233	35.63	0.002
	No	544	72.15	421	64.37	
Systemic condition						
	Yes	195	25.86	138	21.1	0.036
	No	559	74.14	516	78.9	
Nutritional condition (BMI)						
	Underweight	6	0.8	24	3.67	<0.001
	Normal weight	363	48.14	398	60.86	
	Overweight	281	37.27	191	29.2	
	Obesity	104	13.79	41	6.27	
Academic level at which they work						
	Undergraduate	24	9.72	115	23.09	<0.001
	Postgraduate	194	78.54	361	72.49	
	Undergraduate and postgraduate	29	11.74	22	4.42	
Role						
	Administrative and auxiliar staff	179	67.24	156	23.85	<0.001
	Student	481	20.29	432	66.06	
	Faculty	94	12.47	66	10.09	
Place						
	San Isidro	58	7.69	29	4.43	<0.001
	SMP	585	77.59	386	59.02	
	San Isidro and SMP	111	14.72	239	36.54	
Provenance						
	Lima	652	86.47	616	94.19	<0.001
	Provinces	102	13.53	38	5.81	
Gender						
	Male	410	54.38	211	32.26	<0.001
	Female	344	45.62	443	67.74	
Age						
	Young (18–29 years old)	151	20.03	280	42.81	<0.001
	Adult (30–59 years old)	576	76.39	339	51.83	
	Elder (60 years old and over)	27	3.58	35	5.35	
Total	1408	754	53.55	654	46.45	

* Chi-square test.

**Table 2:** Diagnosis, diagnosis methods, and type of clinical spectrum of the COVID-19 epidemiological surveillance plan in an integrated teaching and care service in 2021.

Variables		Year 2021																						
		COVID-19 Diagnosis					Method of COVID-19 Diagnosis									Type of COVID-19 Clinical Spectrum								
		Yes		No		*p* *	Clinical Suspicion		Serological Test		Antigen Test		Molecular Test		*p* *	Asymptomatic		Mild		Moderate		Severe		*p* *
		n	%	n	%		n	%	n	%	n	%	n	%		n	%	n	%	n	%	n	%	
COVID-19 vaccine (at least two doses)																								
	Yes	57	16.06	298	83.94	0.004	12	21.05	19	33.33	6	10.53	20	35.09	0.309	23	40.35	22	38.60	12	21.05	0	0.00	0.317
	No	98	24.56	301	75.44		11	11.22	36	36.73	17	17.35	34	34.69		32	32.65	32	32.65	33	33.67	1	1.02	
Contact with COVID-19 patient																								
	Yes	97	46.19	113	53.81	<0.001	18	18.56	12	12.37	21	21.65	46	47.42	<0.001	10	10.31	44	45.36	42	43.30	1	1.03	<0.001
	No	58	10.66	486	89.34		5	8.62	43	74.14	2	3.45	8	13.79		45	77.59	10	17.24	3	5.17	0	0.00	
Systemic condition																								
	Yes	59	30.26	136	69.74	<0.001	11	18.64	6	10.17	17	28.81	25	42.37	<0.001	3	5.08	32	54.24	24	40.68	0	0.00	<0.001
	No	96	17.17	463	82.83		12	12.50	49	51.04	6	6.25	29	30.21		52	54.17	22	22.92	21	21.88	1	1.04	
Nutritional condition (BMI)																								
	Underweight	0	0.00	6	100.00	<0.001	0	0.00	0	0.00	0	0.00	0	0.00	<0.001	0	0.00	0	0.00	0	0.00	0	0.00	<0.001
	Normal weight	54	14.88	309	85.12		12	22.22	14	25.93	5	9.26	23	42.59		15	27.78	18	33.33	20	37.04	1	1.85	
	Overweight	55	19.57	226	80.43		11	20.00	13	23.64	1	1.82	30	54.55		11	20.00	19	34.55	25	45.45	0	0.00	
	Obesity	46	44.23	58	55.77		0	0.00	28	60.87	17	36.96	1	2.17		29	63.04	17	36.96	0	0.00	0	0.00	
Academic level at which they work																								
	Undergraduate	2	8.33	22	91.67	0.240	0	0.00	0	0.00	0	0.00	2	100.00	0.134	0	0.00	1	50.00	1	50.00	0	0.00	0.458
	Postgraduate	41	21.13	153	78.87		12	29.27	10	24.39	7	17.07	12	29.27		15	36.59	21	51.22	5	12.20	0	0.00	
	Undergraduate and postgraduate	4	13.79	25	86.21		0	0.00	3	75.00	0	0.00	1	25.00		2	50.00	2	50.00	0	0.00	0	0.00	
Role																								
	Administrative and auxiliar staff	38	21.23	141	78.77	0.219	4	10.53	15	39.47	5	13.16	14	36.84	0.013	13	34.21	10	26.32	14	36.84	1	2.63	0.064
	Student	107	22.25	374	77.75		38	35.51	25	23.36	16	14.95	28	26.17		38	35.51	54	50.47	15	14.02	0	0.00	
	Faculty	13	13.83	81	86.17		0	0.00	5	38.46	2	15.38	6	46.15		5	38.46	7	53.85	1	7.69	0	0.00	
Place																								
	San Isidro	19	32.76	39	67.24	0.020	5	26.32	8	42.11	2	10.53	4	21.05	0.059	5	26.32	13	68.42	1	5.26	0	0.00	0.006
	SMP	120	20.51	465	79.49		12	10.00	43	35.83	19	15.83	46	38.33		45	37.50	32	26.67	42	35.00	1	0.83	
	San Isidro and SMP	16	14.41	95	85.59		6	37.50	4	25.00	2	12.50	4	25.00		5	31.25	9	56.25	2	12.50	0	0.00	
Provenance																								
	Lima	124	19.02	528	80.98	0.008	22	17.74	26	20.97	23	18.55	53	42.74	<0.001	25	20.16	53	42.74	45	36.29	1	0.81	<0.001
	Provinces	31	30.39	71	69.61		1	3.23	29	93.55	0	0.00	1	3.23		30	96.77	1	3.23	0	0.00	0	0.00	
Gender																								
	Male	65	15.85	345	84.15	<0.001	12	18.46	35	53.85	1	1.54	17	26.15	<0.001	32	49.23	17	26.15	15	23.08	1	1.54	0.011
	Female	90	26.16	254	73.84		11	12.22	20	22.22	22	24.44	37	41.11		23	25.56	37	41.11	30	33.33	0	0.00	
Age																								
	Young (18–29 years old)	22	14.57	129	85.43	0.002	10	45.45	6	27.27	3	13.64	3	13.64	<0.001	10	45.45	12	54.55	0	0.00	0	0.00	0.011
	Adult (30–59 years old)	133	23.09	443	76.91		13	9.77	49	36.84	20	15.04	51	38.35		45	33.83	42	31.58	45	33.83	1	0.75	
	Elder (60 years old and over)	0	0.00	27	100.00		0	0.00	0	0.00	0	0.00	0	0.00		0	0.00	0	0.00	0	0.00	0	0.00	
Total		754																						

* Chi-square test.

**Table 3:** Diagnosis, diagnosis methods, and type of clinical spectrum of the COVID-19 epidemiological surveillance plan in an integrated teaching and care service in 2022.

Variables		Year 2022																						
		COVID-19 Diagnosis					Method of COVID-19 Diagnosis									Type of COVID-19 Clinical Spectrum								
		Yes		No		*p* *	Clinical Suspicion		Serological Test		Antigen Test		Molecular Test		*p* *	Asymptomatic		Mild		Moderate		Severe		*p* *
		n	%	n	%		n	%	n	%	n	%	n	%		n	%	n	%	n	%	n	%	
COVID-19 vaccine (at least two doses)																								
	Yes	187	30.26	431	69.74	0.752	39	20.86	25	13.37	45	24.06	78	41.71	<0.001	45	24.06	89	47.59	51	27.27	2	1.07	0.006
	No	10	27.78	26	72.22		0	0	7	70	0	0	3	30		7	70	0	0	3	30	0	0.00	
Contact with COVID-19 patient																								
	Yes	148	63.52	85	36.48	<0.001	35	23.65	17	11.49	36	24.32	60	40.54	0.004	29	19.59	68	45.95	49	33.11	2	1.35	<0.001
	No	49	11.64	372	88.36		4	8.16	15	30.61	9	18.37	21	42.86		23	46.94	21	42.86	5	10.2	0	0.00	
Systemic condition																								
	Yes	55	39.86	83	60.14	0.005	7	12.73	6	10.91	19	34.55	23	41.82	0.049	8	14.55	29	52.73	16	29.09	2	3.64	0.016
	No	142	27.52	374	72.48		32	22.54	26	18.31	26	18.31	58	40.85		44	30.99	60	42.25	38	26.76	0	0.00	
Nutritional condition (BMI)																								
	Underweight	1	4.17	23	95.83	0.001	1	100	0	0	0	0	0	0	0.002	0	0	1	100	0	0	0	0	0.171
	Normal weight	111	27.89	287	72.11		26	23.42	8	7.21	27	24.32	50	45.05		21	18.92	51	45.95	37	33.33	2	1.8	
	Overweight	66	34.55	125	65.45		11	16.67	16	24.24	12	18.18	27	40.91		24	36.36	27	40.91	15	22.73	0	0	
	Obesity	19	46.34	22	53.66		1	5.26	8	42.11	6	31.58	4	21.05		7	36.84	10	52.63	2	10.53	0	0	
Academic level at which they work																								
	Undergraduate	50	43.48	65	56.52	0.001	16	32	5	10	14	28	15	30	0.069	10	20	32	64	8	16	0	0	0.046
	Postgraduate	94	26.04	267	73.96		16	17.02	6	6.38	19	20.21	53	56.38		27	28.72	40	42.55	27	28.72	0	0	
	Undergraduate and postgraduate	9	40.91	13	59.09		3	33.33	1	11.11	3	33.33	2	22.22		0	0	7	77.78	2	22.22	0	0	
Role																								
	Administrative and auxiliar staff	44	28.21	112	71.79	0.739	4	9.09	20	45.45	9	20.45	11	25	<0.001	15	34.09	10	22.73	17	38.64	2	4.55	0.001
	Student	131	30.32	301	69.68		31	23.66	10	7.63	32	24.43	58	44.27		33	25.19	63	48.09	35	26.72	0	0	
	Faculty	22	33.33	44	66.67		4	18.18	2	9.09	4	18.18	12	54.55		4	18.18	16	72.73	2	9.09	0	0	
Place																								
	San Isidro	12	41.38	17	58.62	<0.001	0	0	1	8.33	5	41.67	6	50	0.119	1	8.33	9	75	1	8.33	1	8.33	<0.001
	SMP	150	38.86	236	61.14		27	18	26	17.33	33	22	64	42.67		38	25.33	60	40	51	34	1	0.67	
	San Isidro and SMP	35	14.64	204	85.36		12	34.29	5	14.29	7	20	11	31.43		13	37.14	20	57.14	2	5.71	0	0	
Provenance																								
	Lima	180	29.22	436	70.78	0.043	36	20	24	13.33	42	23.33	78	43.33	0.003	42	23.33	86	47.78	50	27.78	2	1.11	0.013
	Provinces	17	44.74	21	55.26		3	17.65	8	47.06	3	17.65	3	17.65		10	58.82	3	17.65	4	23.53	0	0	
Gender																								
	Male	59	27.96	152	72.04	0.406	7	11.86	18	30.51	10	16.95	24	40.68	0.002	18	30.51	31	52.54	9	15.25	1	1.69	0.091
	Female	138	31.15	305	68.85		32	23.19	14	10.14	35	25.36	57	41.3		34	24.64	58	42.03	45	32.61	1	0.72	
Age																								
	Young (18–29 years old)	72	25.71	208	74.29	0.061	27	37.5	5	6.94	20	27.78	20	27.78	<0.001	19	26.39	44	61.11	9	12.5	0	0	<0.001
	Adult (30–59 years old)	116	34.22	223	65.78		10	8.62	27	23.28	23	19.83	56	48.28		31	26.72	40	34.48	44	37.93	1	0.86	
	Elder (60 years old and over)	9	25.71	26	74.29		2	22.22	0	0	2	22.22	5	55.56		2	22.22	5	55.56	1	11.11	1	11.11	
Total		654																						

* Chi-square test.

**Table 4:** Factors associated with the diagnosis, diagnosis methods, and type of clinical spectrum of the COVID-19 epidemiological surveillance plan in an integrated teaching and care service.

Variables		COVID-19 Diagnosis: Yes						Method of COVID-19 Diagnosis: Molecular Test						Type of COVID-19 Clinical Spectrum: Moderate–Severe					
		PR	95% CI	*p*	aPRa	95% CI	*p*	PR	95% CI	*p*	aPRa	95% CI	*p*	PR	95% CI	*p*	aPRa	95% CI	*p*
Year																			
	2021	Ref.			Ref.			Ref.			Ref.			Ref.			Ref.		
	2022	1.47	1.22-1.76	<0.001	1.51	1.10–2.07	0.010	1.25	0.97–1.62	0.083	1.35	0.91–2.00	0.131	0.96	0.96–1.33	0.797	1.06	0.57–1.98	0.862
COVID-19 Vaccine (at least two doses)																			
	Yes	Ref.			Ref.			Ref.			Ref.			Ref.			Ref.		
	No	0.99	0.81-1.21	0.920	1.14	0.61–2.14	0.681	0.75	0.56–1.00	0.052	0.27	0.04–1.78	0.175	1.29	0.92–1.80	0.140	1.12	0.75–1.66	0.584
Contact with COVID-19 patient																			
	Yes	Ref.			Ref.			Ref.			Ref.			Ref.			Ref.		
	No	0.20	0.17-0.24	<0.001	0.20	0.14–0.27	<0.001	0.54	0.39–0.75	<0.001	1.20	0.91–1.58	0.188	0.20	0.10–0.39	<0.001	0.48	0.24–0.96	0.036
Systemic condition																			
	Yes	Ref.			Ref.			Ref.			Ref.			Ref.			Ref.		
	No	0.65	0.54-0.78	<0.001	0.88	0.64–1.22	0.450	0.90	0.70–1.16	0.401	1.20	0.81–1.78	0.366	0.68	0.49–0.95	0.022	1.03	0.50–2.13	0.945
Nutritional condition (BMI)																			
	Underweight–Normal weight	Ref.			Ref.			Ref.			Ref.			Ref.			Ref.		
	Overweight–Obesity	1.44	1.20-1.72	<0.001	1.00	0.76–1.31	0.978	0.66	0.52–0.85	0.001	0.97	0.67–1.42	0.888	0.63	0.45–0.87	0.006	0.80	0.43–1.51	0.497
Academic level at which they work																			
	Undergraduate	Ref.						Ref.						Ref.					
	Postgraduate	0.65	0.50-0.84	0.001	-	-	-	1.29	0.88–1.88	0.191	-	-	-	1.37	0.70–2.67	0.355	-	-	-
	Undergraduate and postgraduate	0.68	0.41-1.14	0.145	-	-	-	0.64	0.23–1.74	0.378	-	-	-	0.89	0.22–3.63	0.870	-	-	-
Role																			
	Administrative and auxiliar staff	Ref.						Ref.						Ref.					
	Student	1.23	1.02-1.49	0.033	-	-	-	1.51	1.15–1.98	0.003	-	-	-	0.63	0.45–0.87	0.006	-	-	-
	Faculty	0.95	0.69-1.32	0.777	-	-	-	1.59	1.10–2.31	0.014	-	-	-	0.22	0.07–0.66	0.007	-	-	-
Place																			
	San Isidro	Ref.						Ref.						Ref.					
	SMP	0.78	0.58-1.05	0.105	-	-	-	1.24	0.75–2.05	0.407	-	-	-	3.64	1.23–10.79	0.020	-	-	-
	San Isidro and SMP	0.41	0.28-0.60	<0.001	-	-	-	1.18	0.64–2.18	0.593	-	-	-	0.81	0.19–3.38	0.773	-	-	-
Provenance																			
	Lima	Ref.						Ref.						Ref.					
	Provinces	1.43	1.11-1.84	0.005	-	-	-	0.17	0.07–0.44	<0.001	-	-	-	0.26	0.10–0.67	0.005	-	-	-
Gender																			
	Male	Ref.						Ref.						Ref.					
	Female	1.45	1.20-1.76	<0.001	-	-	-	1.30	0.99–1.72	0.063	-	-	-	1.59	1.08–2.34	0.019	-	-	-
Age																			
	Young (18–29 years old)	Ref.						Ref.						Ref.					
	Adult (30–59 years old)	1.25	1.01-1.54	0.037	-	-	-	1.17	0.83–1.66	0.363	-	-	-	3.82	2.01–7.26	<0.001	-	-	-
	Elder (60 years old and over)	0.67	0.36-1.25	0.205	-	-	-	1.77	1.01–3.11	0.048	-	-	-	2.32	0.59–9.14	0.229	-	-	-

PR: prevalence ratio. aPR: adjusted prevalence ratio. 95% CI: 95% confidence interval. *p*: statistical significance. a: adjusted by academic level at which they work, role, place, provenance, gender, and age. N = 1408.

## Data Availability

Data are contained within the article.
